# Dexosomes as a cell-free vaccine for cancer immunotherapy

**DOI:** 10.1186/s13046-020-01781-x

**Published:** 2020-11-23

**Authors:** Sepideh Nikfarjam, Jafar Rezaie, Fatah Kashanchi, Reza Jafari

**Affiliations:** 1 Department of Medical Biotechnology, Faculty of Advanced Medical Sciences, Tabriz University of Medical Sciences, Tabriz, Iran; 2 Solid Tumor Research Center, Cellular and Molecular Medicine Research Institute, Urmia University of Medical Sciences, P.O. Box: 1138, Shafa St, Ershad Blvd., 57147 Urmia, Iran; 3 School of Systems Biology, Laboratory of Molecular Virology, George Mason University, Discovery Hall Room 182, 10900 University Blvd., VA 20110 Manassas, USA; 4 Department of Immunology and Genetics, School of Medicine, Urmia University of Medical Sciences, Urmia, Iran

**Keywords:** Exosome, Dexosome, Dendritic cell-derived exosome, Extracellular vesicle, Anti-cancer vaccine, Immunotherapy

## Abstract

Dendritic cells (DCs) secrete vast quantities of exosomes termed as dexosomes. Dexosomes are symmetric nanoscale heat-stable vesicles that consist of a lipid bilayer displaying a characteristic series of lipid and protein molecules. They include tetraspanins and all established proteins for presenting antigenic material such as the major histocompatibility complex class I/II (MHC I/II) and CD1a, b, c, d proteins and CD86 costimulatory molecule. Dexosomes contribute to antigen-specific cellular immune responses by incorporating the MHC proteins with antigen molecules and transferring the antigen-MHC complexes and other associated molecules to naïve DCs. A variety of ex vivo and in vivo studies demonstrated that antigen-loaded dexosomes were able to initiate potent antitumor immunity. Human dexosomes can be easily prepared using monocyte-derived DCs isolated by leukapheresis of peripheral blood and treated ex vivo by cytokines and other factors. The feasibility of implementing dexosomes as therapeutic antitumor vaccines has been verified in two phase I and one phase II clinical trials in malignant melanoma and non small cell lung carcinoma patients. These studies proved the safety of dexosome administration and showed that dexosome vaccines have the capacity to trigger both the adaptive (T lymphocytes) and the innate (natural killer cells) immune cell recalls. In the current review, we will focus on the perspective of utilizing dexosome vaccines in the context of cancer immunotherapy.

## Background

Dendritic cells (DCs) are adept antigen-presenting cells (APCs) of the mammalian immune system that function as the link between innate and adaptive immunity by recognizing, ingesting, processing, and presenting antigenic material to T lymphocytes, leading to either initiation or repression of immune responses [1]. The presentation of the antigenic material is conducted through the major histocompatibility complex (MHC) class I and II molecules to naïve cytotoxic T lymphocytes (CTLs, CD8^+^ T cells) and naïve helper T cells (Ths, CD4^+^ T cells), respectively. DCs are a heterogeneous subpopulation of immune cells that are produced from precursor cells like monocytes in the bone marrow and are distributed among all organs and tissues via blood circulation. Upon antigen recognition, DCs start to travel through lymphatic vessels to the T cell zones of lymphoid tissues. Throughout this journey, DCs are matured and express costimulatory molecules and when reached their destination, they discern and stimulate their cognate T lymphocytes [1].

DCs exert pivotal functions in inducing protective immune responses throughout pathological conditions, e.g. oncogenesis, since they are able to recognize tumor-associated antigens (TAAs). DC-primed CD8^+^ CTLs are able to identify TAAs incorporated with MHC I proteins on the cellular membrane of cancer cells and destroy them [2]. Naïve CD4^+^ T cells are on the other hand differentiated into effector cells which initiate B cell-related TAA-specific antibody responses. In the meantime, DCs also have the potential to induce T cell anergy and develop a cancer-promoting local microenvironment through expressing immune checkpoint molecules and releasing anti-inflammatory cytokines like transforming growth factor β (TGFβ) and interleukin 10 (IL10) [3]. Since cancer cells often lose the potential of undergoing programmed cell death [4], activating the host immune system through TAA-loaded DC-based vaccines is one of the suggested approaches in cancer immunotherapy to eradicate tumor cells. To date, sipuleucel-T (Provenge, Dendreon Corporation) has been the only DC vaccine that was granted the approval of the US Food and Drug Administration for therapy of asymptomatic metastatic castration-resistant prostate cancer (mCRPC) [5]. The autologous DCs in sipuleucel-T were activated using a recombinant fusion protein containing prostatic acid phosphatase (PAP, a prostate TAA) and granulocyte-macrophage colony-stimulating factor (GM-CSF). Sipuleucel-T therapy was associated with an improvement in overall survival of four months in comparison with the participants of the placebo control group [5].

The production of sufficient DCs for preparing cancer vaccines can be challenging. The shifting molecular composition of DCs renders obstacles in vaccine quality control and the low abundance of TAA-MHC II complexes on DC surface results in lower yields. According to clinical investigations, DC vaccines rely mainly on chemotactic signaling to access and localize in lymph nodes and were unsuccessful to elicit pro-natural killer cell (NK) effects due to the low expression of NK receptor ligands. Furthermore, DC vaccines are susceptible to immunosuppressive molecules and signals present in tumor microenvironment [6]. Therefore, a novel platform for more efficient delivery of high levels of TAAs concomitant with co-stimulatory factors have been utilized in the recent preclinical and clinical studies, called dexosome vaccines.

DC-derived exosomes or dexosomes are small lipid vesicles released from DCs that have received immune signals. Within an activated DC, dexosomes incorporate the processed peptides derived from antigenic material with MHC I/II on their surface and deliver the functional peptide-MHC complexes to distal naïve DCs. As a result, the target DCs will be stimulated and acquire the competency to trigger cognate T cells [7]. Therefore, dexosomes function as vehicles that disseminate antigenic material amongst DCs, exerting a noble mechanism designed for immune response amplification. This theory is the major rationale for utilization of dexosomes as vaccine tools in cancer immunotherapy. Dexosomes present 10 to 100 folds more TAA-MHC II complexes as compared to DCs [6]. Moreover, the molecular composition of dexosomes can be precisely defined for each donor patient. Due to the stability of dexosomal lipid composition, cryopreservation of the vaccine preparation is possible for longer than six months at − 80 °C. Once injected, dexosome vaccines are easily dispersed within lymph nodes and can access to a variety of immune cells, and their trafficking and localization is not reliant on chemokines but rather on their surface receptor topography. More importantly, dexosomes express ligands for NK receptors and are not influenced by the immunosuppressive tumor microenvironment. The aim of this review was to discuss: (i) diverse subsets of DCs and their specific role in tumor microenvironment; (ii) exosomes and their biogenesis process; (iii) dexosomes and how their function leads to activation of cognate T cells; (iv) how DC status affects dexosome release; (v) the potential therapeutic implications of dexosomes in preclinical studies and clinical trials; and (vi) the future direction of dexosome-based vaccines in cancer immunotherapy.

### Different subsets of DCs

DCs are a heterogeneous subpopulation of immune cells that are grouped into various subsets, both in mouse and human, according to their ontogeny, phenotype, tissue localization, molecular composition, and biological function [8, 9]. Conventional DCs (cDCs), plasmacytoid DCs (pDCs) and monocyte-derived DCs (moDCs) comprise the three classic subsets of DCs in human [8, 9]. cDCs are subdivided into cDCs type 1 and 2 (cDC1s and cDC2s) based on the repertoire of transcription factors that regulate their development. Whereas IRF8 (IFN regulatory factor 8), the DNA-binding protein inhibitor ID2, and BATF3 (basic leucine zipper transcriptional factor ATF-like 3) control the development of cCD1s, IRF4, ID2, ZEB (zinc finger E-box-binding homeobox protein), and Notch2/krueppel-like factor 4 (KLF4) regulate the development of cCD2s [10]. Both subgroups of cDCs exhibit different phenotypical and functional characteristics. cDC1s are CD141/BDCA3^+^ cells that were shown to express the C-type lectin receptor DNGR1/CLEC9A and the chemokine receptor XCR1 [11–13]. Moreover, they are involved in cross-presenting antigen-MHC I complexes to CD8^+^ T cells. On the other hand, cDC2s (CD1c^+^ cells) produce CD172a (a signal regulatory protein) and contribute to cross-presentation of antigen-MHC II complexes to CD4^+^ T cells [14]. pDCs represent an additional subset of DCs that are known for expressing CD123, BDCA2, and BDCA4 proteins and generating interferon (IFN) type I molecules [14, 15]. moDCs are absent under homeostatic conditions, but during inflammation, they are developed from monocytes and travel to the inflamed regions and trigger the polarization of CD4^+^ T cells [1, 16]. Additional subsets of DCs have also been defined based on the data obtained from high-throughput single-cell RNA sequencing [17].

Because of the various migratory features and tissue positioning of different DC subsets, DC biology is very complicated [18, 19]. During certain pathological contexts, such as tumorigenesis, particular subsets of DCs are recruited and each subset plays pivotal roles in exerting antitumor immune responses by induction of certain T cell subsets through expressing costimulatory factors and pro-inflammatory cytokines [20]. Thereby, DCs may present novel instructions for creating potent and efficient protective immunity against tumor cells [21, 22].

### Anti-tumor functions of different DC subsets in tumor microenvironment

#### cDC1s

Cross-presentation of TAAs through DCs is mandatory for effective stimulation of T cells and initiation of antitumor cytotoxic effects [23]. cDC1s are dedicated to incorporate TAAs into MHC I proteins and present the TAA-MHC I complexes to CD8^+^ CTLs [24, 25]. Certain proteins associated with membrane trafficking are necessary for this process, including Sec22b (a member of the soluble N-ethyl maleimide (NEM)-sensitive factor attachment protein receptor (SNARE) proteins) and WDFY4. These proteins are not only necessary for controlling tumor growth but also they are required for the effectiveness of immunotherapies based on anti-PD1 (programmed cell death protein 1) agents [26, 27]. In addition to the proteins involved in TAA cross-presentation, there are other cDC1-associated proteins required for promotion of antitumor immune recalls [28]. For efficient stimulation of CD8^+^ T cells, TAAs should be transferred to lymph nodes draining the tumor via migrating CD103^+^ cDC1s in a CCR7-restricted mode [29]. XCR1 expressed by cDC1s contributes to the development of antitumor immunity by orchestrating localization of DCs in response to XCL1 (the XCR1 ligand) expressed by CTLs and NKs [30, 31]. cDC1s also promote local antitumor immune responses via producing CXCL9 and CXCL10 chemokines that stimulate CXCR3^+^ effector T cells and NKs [32, 33]. Moreover, these chemokines coordinate the localization of memory CD8^+^ T cells in cDC1-abundant regions to improve local restimulation of T cells [34, 35]. cDC1s also produce and release a large amount of IL12 which stimulates CTL and NK cytotoxic activity and promotes production of IFNγ [36–39]. NKs were shown to have the ability of employing circulatory cDC1s to nearby tissues and tumors [40]. Flt3L molecules produced from intratumor cDC1s preserve the viability and activity of cDC1s inside the tumor micro environment and trigger local differentiation of DCs from their precursor cells [41]. Along with the induction of CTL expansion, cDC1s are also capable of stimulating the production of CD4^+^ Th1 cells by cross-presenting TAA-MHC II complexes [42]. The antitumor activity of cDC1s is supposed to be further assisted by pDCs [43]. As main producers of type I IFN, pDCs trigger antigen cross-presentation and CD8^+^ CTL antitumor immune response [44, 45]. Taken together, cDC1s represent an effective system for antitumor CTL stimulation by interacting with components of both the innate and adaptive immune systems.

#### cDC2s

Due to the absence of selective membrane markers required for precise detection of cDC2s in pathological contexts and the unavailability of sufficient preclinical investigations, the function of cDC2s in cancer immunology remains to be fully elucidated. cDC2s contribute to cross-presentation of TAA-MHC II complexes to CD4^+^ T cells [46–50]. As a result, the activated CD4^+^ T cells promote antitumor immune responses by secreting IFNγ that triggers macrophages and NKs, blocks angiogenesis, regulates the tumor stroma formation, and leads to direct tumor cell lysis [51]. However, compared to cDC1s, cDC2s are less potent in cross-processing TAAs, migrating to tumor-draining lymph nodes, secreting IL12, and activating CD8^+^ CTLs [29, 32, 36, 52].

The communication and interaction between DC subsets and T cells plays a critical role at different stages of antitumor immunity. Maximal stimulation of CTLs is dependent on the activation of both cDC1s and cDC2s [42, 51, 53]. It was demonstrated that cDC2s lose their ability to induce CD4^+^ T cell differentiation during tumor growth. However, when T regulatory cells (Tregs) were depleted, cDC2 migration and activation capacity of CD4^+^ T cells for producing IFNγ was enhanced [54]. Additionally, cDC2s were reported to activate CD4^+^ T cells toward IL17-producing T cells [46].

Studies have shown that the functions of cDC1s and cDC2s may overlap to some extent, for example they both produce IL12 and depend on Flt3L for their development [55, 56]. Moreover, the number of blood-borne cDC1s and cDC2s is generally reduced in cancer patients [57]. However, cDC2s are known for not having a unique pattern of gene expression. Rather, they demonstrate a common gene expression pattern with monocytes with only a number of genes preferentially expressed, e.g. *CCL22* which encodes for a chemokine that activates CCR4^+^ T cells [58]. It was shown that tumor-associated cDC2s possess langerin-encoding *CD207* gene as a marker both in human and mouse lung tumors [59].

#### pDCs

pDCs participate in exerting protective antitumor immune responses by producing IFNα that inhibits tumor growth, angiogenesis, and metastasis [60]. Both ex vivo and in vivo models [61, 62] demonstrated the direct cytotoxic function of pDCs via producing and secreting Granzyme B and TRAIL (TNF-related apoptosis-inducing ligand) molecules [63, 64]. pDCs are also capable of exerting indirect antitumor immunity by the OX40L-mediated production of IFNγ and the CCR5-mediated recruitment of NKs [65]. A unique subset of pDCs were identified in head and neck squamous carcinoma that overexpress OX40 and was reported to demonstrate synergizing effects with cDCs in inducing effective TAA-specific CD8^+^ T cell responses [66].

moDCs.

Due to their overlapping functions of moDCs with other myeloid cells, their role in exerting antitumor immunity in human is not clear yet. However, they probably play significant roles in stimulating the propagation of naïve CD8^+^ T cells [67]. Preclinical investigations suggested central roles for moDCs in regulating antitumor immunity during chemotherapy, cell vaccination, and T cell adoptive therapy [68–70].

### Exosomes

Extracellular vesicles (EVs) are classified into three main groups according to their origin and size: exosomes (30–150 nm in diameter), apoptotic bodies and microvesicles or shedding particles (both larger than 100 nm). Microvesicles and apoptotic bodies are constructed by direct sprouting of the cellular membrane in living and dying cells, respectively. Exosomes, on the other hand, are formed by inward budding as intraluminal vesicles (ILVs) within the lumen of multivesicular bodies (MVBs, or so-called late endosomes). Once the MVB fuses with the cellular membrane, these ILVs are secreted to the extracellular space as free exosomes [71]. It was initially presumed that exosomes were an alternate route to excrete waste products in order to sustain cellular homeostasis. Today, however, it is well established that exosomes play significant roles in intercellular communication and were reported to be correlated with a variety of physiological and pathological conditions.

As a general rule, the composition of exosomes partially mirrors the composition of the donor MVBs and thus the parent cells. The nature and the abundance of exosomal cargos depend on the cell type and state, the stimuli that tune the construction and secretion of exosomes, and the molecular pathways that mediate their biogenesis [72]. Exosomal proteins belong to distinct functional groups. These include cell adhesion molecules (CAMs) including tetraspanins, integrins, and milk fat globule-EGF factor 8 protein (MFGE8, lactadherin), antigen presentation molecules (MHC I and II and costimulatory molecules such as CD86), membrane transport and fusion proteins like annexins and RAP1B/RABGDI, Rab 2 and 7, heat shock proteins (HSPs), cytoskeletal proteins, raft-associated proteins and glycolipids, pyruvate kinase and alpha enolase enzymes, and other proteins inclusive of elongation factor 1α, clathrin, ferritin, and the ESCRT (endosomal sorting complexes required for transport) proteins Alix and Tsg101 [73]. While the protein content may vary among different exosomes, the exosomal lipid composition is generally conserved and cell type-specific. The high density of lysobisphosphatidic acid in the internal lipid layer of MVB membrane facilitates the inward budding of MVBs and thus exosome formation through interacting with Alix [74]. Exosomes can influence the homeostasis of their recipient cells by altering their lipid profile particularly in cholesterol and sphingomyelin [74].

### Biogenesis of exosomes

**Fig. 1:**
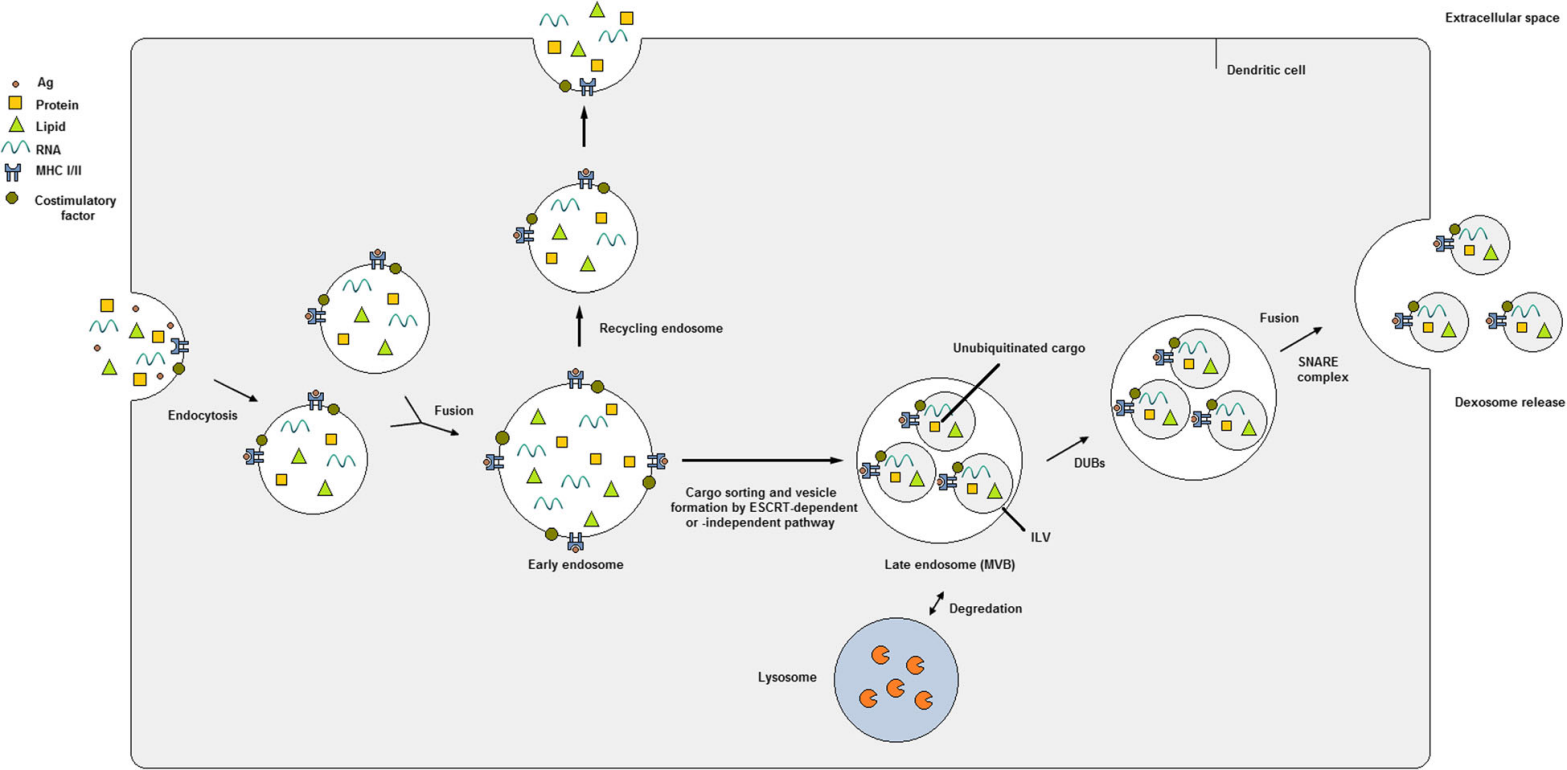
Dexosome generation and release within the endosomal system of dendritic cells (DCs). Endocytic vesicles including a variety of extracellular and membrane cargos join together to form early endosomes (EEs). Now EEs can follow two pathways: either returning to the plasma membrane as recycling endosomes or transformation into late endosomes (LEs) or so-called multivesicular bodies (MVBs). Within MVBs, the lipid membrane starts to sprout inwardly concomitant with packing of the ubiquitinated cargos into the nascent intraluminal vesicles (ILVs). MVB membrane budding and cargo sorting of ILVs can be conducted using either ESCRT-dependent or -independent routes. Later, the generated ILVs are targeted for lysosome degradation unless they are rescued by deubiquitinating enzymes (DUBs). MVBs are then directed toward the DC periphery via cytoskeleton proteins and microtubules, and fuse with the plasma membrane using the SNARE protein components. ILVs are now secreted to the extracellular environment as dexosomes

During the biogenesis of exosomes, cargos are first directed to the location of exosome production at the MVB membrane. Concurrently, the MVB membrane-associated proteins and lipids are gathered as clusters in distinct dynamic platforms, so-called microdomains of the MVB membrane [71, 75]. Exosomal membrane cargos are either internalized from the cellular membrane or obtained from the Golgi apparatus and reach endosomes prior to being sorted into ILV lumens [76]. The crossroad between cargo sorting into MVBs for generation of exosomes and endosomal membrane recycling is regulated by the syntenin protein [77]. Furthermore, a posttranslational ubiquitin-like modification, so-called the ISGylation process, was also recently suggested to play a critical role in controlling MVBs’ fate. The authors proposed that ISGylation of MVB protein components promotes the fusion of MVBs with lysosomes [78]. If the MVBs are destined to form exosomes, the aforementioned membrane microdomains cooperate with other exosome-producing machines and cargos intended for sorting into ILVs and contribute to the invagination of the MVB membrane and formation of small vesicles, followed by fission and releasing of ILVs into the luminal medium in a stepwise manner (Fig. 1). The clustering of exosomal cargos and subsequent sprouting of MVB membrane can be performed by either the ESCRT-dependent or -independent pathways.

The ESCRT apparatus is consisted of several protein complexes, namely ESCRT 0, I, II, III and the associated AAA ATPase Vps4, that function cooperatively in a stepwise manner [79]. Components of the ESCRT 0 and I complexes collect the ubiquitinated transmembrane molecules at the MVB microdomains by recruiting Hrs heterodimer and signal transducing adapter molecule (STAM) 1 and 2. Hrs also associates with Eps15 and clathrin proteins and recruits clathrin to interact with the ubiquitinated cargo [79]. Afterwards, the ESCRT II complex employs the subunits of ESCRT III and the ATPase Vps4 to create sprouts that bud toward the MVB lumen and subsequently the microdomain fission is conducted. The produced ILVs can now be directed for lysosomal degradation unless their cargos are deubiquitinated by DUBs (deubiquitylating enzymes) [80]. The ESCRT machinery is also associated with the ESCRT accessory protein Alix and syntenin that together connect exosomal cargos with the ESCRT III subunit Vps32 (vacuolar protein sorting-associated protein 32) [81].

Studies have revealed that ILVs and thus exosomes can be still generated and released when the main components of the ESCRT protein complexes are silenced or depleted [82]. The first step of the ESCRT-independent pathway for exosome formation is the hydrolysis of sphingomyelin to ceramide that imposes a negative membrane curvature on MVBs [83]. Proteins of the tetraspanin family are among the key modulators of the ESCRT-independent endocytic sorting pathway. Different members of tetraspanins are gathered along with other transmembrane and cytosolic proteins and contribute to formation of clusters and then microdomains that will finally sprout within the MVBs [84]. Several tetraspanin proteins including CD9, CD81 and CD82 were reported to be involved in regulation of exosomal cargo sorting [85]. Another protein playing a significant role in the ESCRT-independent pathway is the SIMPLE protein (the small integral membrane protein of the lysosome/late endosome; also known as LITAF: lipopolysaccharide (LPS)-induced tumor necrosis factor (TNF)). Mutation of the *LITAF* gene inhibited the generation of MVBs whereas the release of exosomes were increased after COS cells were transfected with the *LITAF* [86]. In general, it seems that both the ESCRT-dependent and -independent pathways are highly interconnected and operate in a concerted manner throughout the exosome biogenesis process while overlapping to some extent.

As mentioned before, MVBs are either destined for degradation by lysosomes due to their ubiquitinated content or they may fuse with cellular membrane and release exosomes. In the latter, MVBs are transferred to their final destination in the cell periphery via actins and the associated cytoskeletal proteins and microtubules [87]. Rab27A and B from the Rab GTPase protein family induce the transfer of MVBs [88], and the SNARE protein complex mediates the fusion of MVBs with the plasma membrane and subsequent exosome secretion [74]. First, the calcium-sensing protein, synaptotagmin, localizes on syntaxin (a plasma membrane protein) and the MVB membrane. Now, the collected MVBs can dock the cellular membrane by means of the SNARE complex including three Q-SNARE subcomplexes (typically T-SNARE) on the plasma membrane and one R-SNARE subcomplex (typically V-SNARE) on the MVB, and release exosomes [89]. Once released, exosomes are carried to and captured by the recipient cells where they are either internalized by the cells, fused with the plasma membrane, or stay attached to the cell surface.

### Dexosomes

#### Dexosomal content

Exosomes produced and released by DCs are termed as ‘dexosomes’. As is the case with other exosomes, dexosomes have a characteristic molecular profile of their own. Human dexosomes contain cargos that together operate as a whole antigen-presenting entity. These include a variety of all the known antigen-presenting molecules, such as MHC I/II proteins and costimulatory factors like CD86 [90], which are employed for cross-presentation of peptide antigens to CD8^+^ and CD4^+^ T cells and subsequent triggering of their proliferation. Dexosomes also harbor CD1a, b, c, and d proteins that are involved in cross-presentation of lipid antigens [90]. Dexosomal ICAM1 (intercellular adhesion molecule 1, CD54) was shown to play pivotal role in regulating DC-T cell communication [91]. As a ligand of Mac1 integrins (CD11b/CD18) [92] and the lymphocyte function-associated antigen 1 (LFA1, CD11a/CD18) [93], ICAM1 can either facilitate dexosomal capture by target DCs or promote the interaction of T cells with dexosome-receiving DCs that hold dexosomes on their external surface. Expressing an abundance of microdomain-organizing tetraspanin proteins including CD9, CD37, CD53, CD63, CD81, and CD82, which regulate dexosome-target DC interactions, are considered the hallmark of dexosomes [94]. The presence of CD55 and CD59 molecules on dexosomal surface prevents complement-mediated degradation of dexosomes throughout their extracellular journey [95]. Tsg101 and Alix proteins determine the sorting of ubiquitinated cargos into ILVs during dexosome generation process [96]. MFGE8, that binds to phosphatidylserine on the external surface of dexosomes, promotes dexosomal uptake through interacting with integrins αvβ3 and αvβ5 on APCs [97]. However, successful capture of MFGE8-deficient dexosomes by bone marrow-derived DCs (BMDCs), which produce little or no αvβ3 or αvβ5 integrins in vitro, proved the presence of MFGE8-independent machineries involved in dexosomal uptake [98]. While HSPs, FasL, and CD11b and c are common between human and mouse dexosomes, MFGE8 was only detected in murine monocyte-derived DC (MCDC) dexosomes [98]. HSC73, a member of the HSP70 family, is also abundantly present within the dexosomal cytosol [97]. In cooperation with the members of the HSP90 family, HSC73 probably regulates the immunogenicity of dexosomes by triggering different cells of the immune system and playing pivotal roles in MHC loading and as antigen chaperones [99]. In addition to proteins, dexosomes also harbor various RNA species with the aim of intercellular communication and to induce certain posttranslational modifications in the recipient DCs. For example, it was suggested that dexosomal transfer of miRNAs could suppress the targeted mRNAs in DCs [100], indicating that certain RNA profiles of dexosomes, or particularly those of parent DCs, can impact dexosome immunogenicity.

#### Dexosomal membrane structure

In comparison with the cellular membrane, dexosomal membrane shows an increased transverse diffusion of phospholipids (flip-flop movements) which results in a loss of lipid asymmetry in the membrane structure. The elevated transbilayer movements of phospholipids along with the rigidity of dexosomal membrane at neutral pH control their fusion with other membranes which in result guarantees the stability of dexosomes in circulation [101]. The phospholipid composition of dexosomes is also distinct from their donor DCs. While the amount of sphingomyelin is twice as high in dexosomes, phosphatidylcholine is much lower and cholesterol is absent from dexosomal membrane. A remarkable enrichment of disaturated molecular species such as phosphatidylethanolamines was also distinguished in dexosomal membranes in addition to a 50% decrease in molar ratio of diglicerides:phospholipids. Further investigations demonstrated the abundance of phospholipase D2 in dexosomes, and that phospholipid D probably mediates the putative signaling properties of dexosomes either by cooperating with a second messenger like phosphatidic acid or by association of dexosomes with the target DCs through the fusogenic quality of phosphatidic acid [101, 102].

### Dendritic cell status affects dexosome production and release

While reticulocytes [103], T cells [104], mastocytes [105], and resting B cells [106] secrete exosomes only when a cell surface receptor is triggered, DCs [107], macrophages [108], and most tumor cells constantly release exosomes in vitro. Both mature and immature DCs have the ability to secrete dexosomes, however, the level of dexosome release changes throughout their cellular life cycle. It is assumed that the maturation stage of DCs adversely impacts the extent of dexosome secretion in vitro [91]. According to Thery et al., production of MVB and thus dexosomes is downregulated upon DC maturation, indicating that dexosomes are probably produced by immature DCs in the periphery [97]. Another example is the LPS-matured DCs that release 25–75% less dexosomes compared to immature DCs [97]. Transient increase of dexosome secretion by immature DCs upon cognate interaction with T cell clones indicates that some stimulating signals produced by T cells probably trigger dexosome secretion [109]. On the other hand, a more recent report showed that dexosome release increases upon DC maturation. Here, it was demonstrated that maturation process reformed the molecular make-up of dexosomes and improved their cluster-forming ability, with the latter being associated with the filtration-based technique utilized for dexosome isolation [110]. Dexosomes from immature and mature DCs show distinct bioactivities because of the variations in their protein content. However, controversial findings were reported in this respect. Compared to dexosomes of immature DCs, dexosomes of mature origins were shown to produce greater abundance of MHC I, II, ICAM1, CD80 and CD86 molecules and resembled their donor cells, presenting more immunostimulatory effects [111]. However, a separate set of studies revealed that immature DCs express an average of two- to threefold more dexosomal proteins than mature DCs (0.5 ± 0.1 µg/million immature DC vs. 0.2 ± 0.1 µg/million mature DC) [91]. Because the qualitative variances between dexosomes of immature and mature DC origins were assumed irrelevant at first, the pioneer studies were conducted using mostly dexosomes from immature human or mouse DCs.

Additional factors such as DNA-damaging treatments, like senescence induction or radiation, were also demonstrated to affect the release of dexosome-like vesicles by DCs [112]. In such deleterious treatments, activation of p53 transcription factor upregulates the TSAP6 pathway (the transmembrane protein tumor suppressor-activated pathway 6) which subsequently enhances secretion of dexosomes [112]. Exposure of DCs to various cytokines may alter the phenotype and immunogenicity of released dexosomes. For instance, cultured BMDCs turn into immunosuppressive cells when they are exposed to IL4 and IL10. Dexosomes derived from such immunosuppressive cells were shown to hinder delayed-type hypersensitivity (DTH) and rheumatism in mice most probably by recruiting MHC II and the CD95-CD95L signaling pathway [113]. In another study, only when IFNγ was used for maturation of MCDCs, the NK-activating ligands ULBP1 and IL15Rα were identified on the surface of secreted dexosomes [111]. The use of IL3 and IL4 for maturation of human MCDCs resulted in overexpression of MHC proteins on the dexosomal surface when compared to GM-CSF/IL4-exposed DCs. However, immunogenic properties of the secreted dexosomes remained unaltered in vitro [114]. On the contrary, BMDCs treated with GM-CSF and IL10 released immunosuppressive dexosomes that were able to inhibit inflammation in an arthritis model [115].

### Dexosome function

Dexosomes incorporate whole or partially processed antigen-derived peptides with MHC I/II molecules on their external surface and deliver the functional peptide-MHC complexes to distal cells including APCs. As a result, the target naïve DCs will acquire the competency to stimulate cognate T cells via T cell receptors (TCRs) and initiate adaptive immune responses. This function has been clearly defined by different research groups studying mouse [116], rat [117], and human [118] dexosomes. It is generally assumed that the whole dexosomal protein content is transferred as a complete patch and in a concerted manner to the recipient cells. This way, a preformed functional antigen-stimulating machinery is transported to the target cells which will then become functional in terms of cognate T cell stimulation. Given the fact that a single human DC in culture has the potential of secreting 1 million MHC II molecules per day and only a limited number of peptide-MHC I/II complexes are adequate for stimulating a T lymphocyte [119], the dexosomal pathway is capable of rapidly disseminating and amplifying the cellular immune response.

#### Dexosome-mediated T cell activation: Direct and indirect pathways

**Fig. 2:**
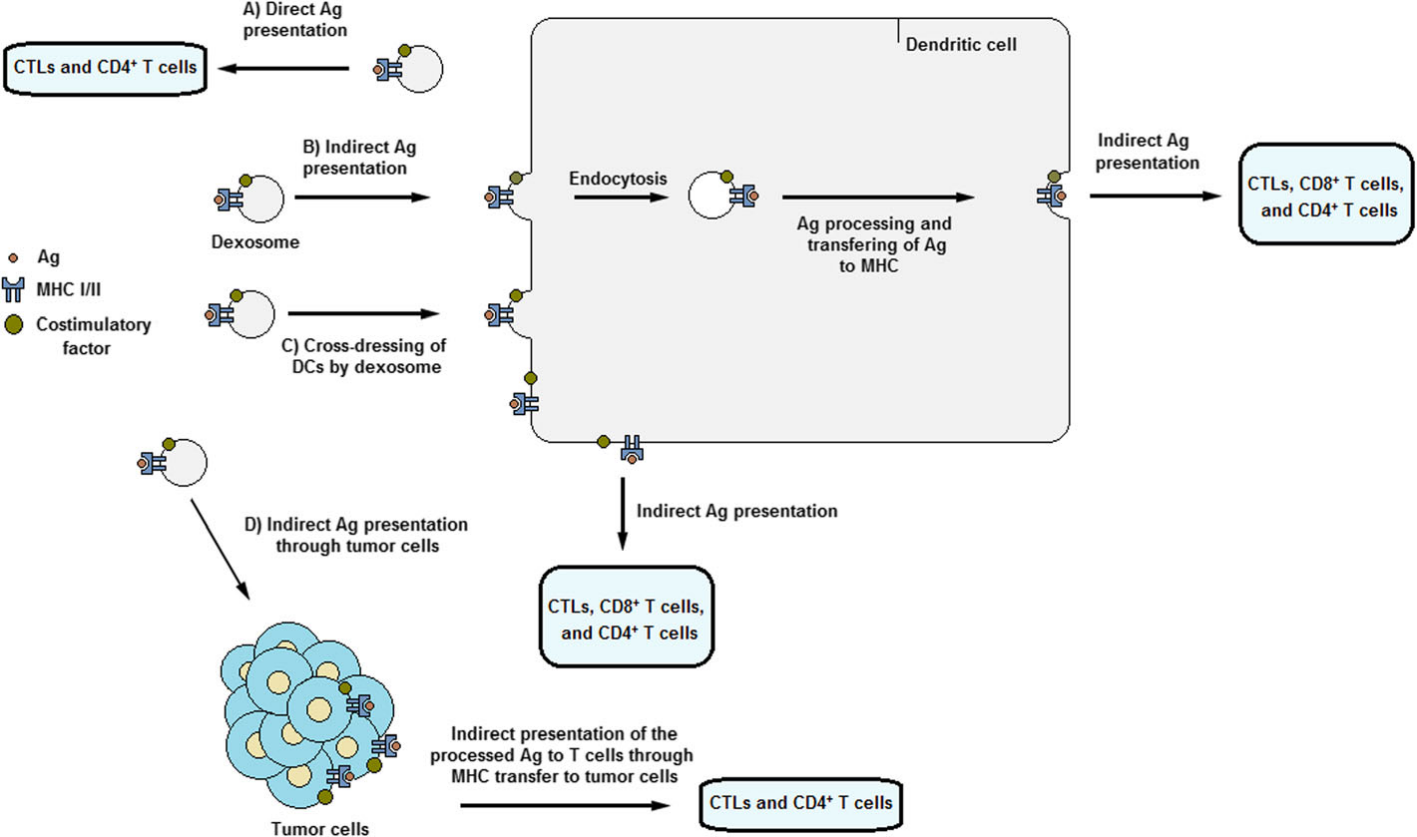
Dexosome function. **a** Direct presentation of antigens by dexosomes to T cells (Antigens are not captured or processed by DC in this pathway). **b** Indirect presentation of antigens by dexosomes to T cells. Here, antigens are first captured and processed by DCs through the endocytic pathway and are subsequently cross-presented on DC surface to T cells. **c** Indirect antigen presentation to DCs through cross-dressing of DCs by dexosomes. **d** Indirect antigen presentation by dexosomes to DCs via tumor cells. Here, antigens, i.e. TAAs, are captured by tumor cells and then cross-presented to T cells

Several mechanisms have been suggested on the topic of how dexosomes contribute to cross-presentation of TAAs by means of MHC proteins and trigger T cell immunity in lymph nodes. One theory suggests direct triggering of T cells by dexosomes in vitro (Fig. 2a). However, the direct dexosome-T cell route was reported unsuccessful in inducing naïve T cells and is less likely to arise extensively in vivo [120]. Most probably, dexosomes are not able to interact with T cells until they are captured by other DCs which extract and process the antigenic material from the TAA-MHC complexes and use them for priming specific T cells [121]. Furthermore, it is more probable that the direct mechanism is only efficient in restimulating memory T cells, formerly-activated T cells, or T cell clones, lines and hybrids [122]. Therefore, dexosomes possess less T cell stimulatory capacity than their parent DCs [117], although their stimulation potency can be promoted when they are immobilized or when their concentration is enhanced [117, 122].

Rather than a direct route for dexosome-T cell activation, accumulating evidence revealed that dexosomes or any other APC-originated exosomes initiate T cell immunity via indirect presentation of antigens to the adjacent APCs [7]. According to this hypothesis, the biological cycle of dexosome release continues as follows (Fig. 2b): immature naïve DCs regularly circulate within the body in order to identify exogenous molecules and antigens originating either from infectious sources or tumors. Once encountered, DCs present at the locality of infection or tumors capture the antigens, e.g. TAAs, and process and convert them to smaller peptide fragments which are then incorporated into MHC I and II molecules. Upon maturation, the antigen-MHC complexes are located on the external surface of DCs and they start to travel to local lymph nodes where they prime antigen-specific T cell responses. Throughout this migration, the antigen-MHC molecules and other immunostimulatory factors are sorted into dexosomes and released to the extracellular space. The secreted dexosomes then proceed to lymph nodes where they transfer their components to the resident naïve DCs. ICAMs and integrins contribute to uptake of dexosomes by bystander DCs. After binding to DCs, some of dexosomes (and not all of them) are internalized and the remaining vesicles probably retain on the external surface of DCs. The fraction of internalized dexosomes depends on the maturation status of the target DCs. Mature DCs maintain dexosomes mostly on their external surface whereas immature DCs tend to internalize them. It is assumed that the surface-retained dexosomes still have the capacity to stimulate T cells [120]. If internalized, the peptide-MHC complexes are processed via the endosomal route, leading to the transfer of dexosome-borne antigenic peptides to MHCs [120, 122, 123]. Afterwards, these antigen-MHC complexes are carried back to the DC membrane surface where they are presented to T cells [90]. Stimulation of naïve T cells was demonstrated to take place only in the presence of APCs [120]. The activation status of parent DCs affects the efficiency of indirect T cell stimulation mechanism to a great extent. For instance, LPS- or IFNγ-matured DCs release dexosomes that abundantly express ICAM1 which improves dexosomal capture, MHC and CD86 molecules which facilitate T cell priming [111].

A second indirect route proposed for dexosome-mediated T cell priming is a process called ‘dexosome-to-DC cross-dressing’ in which dexosomes convey their peptide-MHC molecules to DCs by directly fusing with their plasma membrane (Fig. 2c) [124]. This allows T cells to immediately recognize MHC-incorporated peptides and take advantage of the costimulatory factors and additional molecules on DC surface without the necessity for internalizing or processing of the antigens by DCs. To support this pattern, Thery et al. demonstrated that dexosomes could prime T cells only in the presence of mature CD8α^−^ DCs, even if the mature DCs were MHC II-deficient [125]. This proves the presence of dexosome-to-DC cross-dressing of antigen-MHC molecules which depends greatly on CD80 and CD86 costimulatory molecules for stimulation of T cells [125]. However, dexosome MHC I cross-dressing of adjacent DCs did not trigger the stimulation of ovalbumin-specific CD8^+^ T cells. Instead, dexosomes were internalized (as mentioned before) and the antigen-MHC I complexes were subsequently presented on the DC surface [126].

A third mechanism through which dexosomes can induce T cells occurs via cancer cells (Fig. 2d). Based on recent observations, dexosome-treated human breast adenocarcinoma cells (in comparison with untreated cells) were able to restimulate formerly activated T cells, resulting in extensive proliferation of IFNγ-secreting T cells [127]. Reception of dexosomes by cancer cells indicates the feasibility of converting cancer cells to more powerful immunogenic targets which presents novel avenues for development of therapeutics that enhance tumor immunotargeting.

#### Dexosome function in cancer

In a cancer setting, a significant role of DCs and dexosomes is to identify and destroy tumor cells. Indeed, DCs represent the initial bridge between the host immune system and the ongoing oncogenesis process. This is the first stage of cancer-immunity cycle that attempts to eradicate tumor cells by activating T cells [128]. During oncogenesis, TAAs are produced and secreted and then captured and processed by proximal DCs for cross-presentation to T cells, which results in anti-TAA T cell priming. However, T cell propagation is only stimulated when given additional prerequisites are met within the local tumor microenvironment [128]. These include local immunogenic signals, i.e. proinflammatory cytokines and pathogen- or damage-associated molecular patterns (PAMPs or DAMPs), which force DCs to present the received TAAs to cognate T cells through MHC I/II and costimulatory proteins [128]. Dexosome-based antitumor signaling route is able of modulating tumor cells beyond the level of conventional ligand-receptor signaling routes, leading to elaborate modifications which control tumor progression and antitumor immune responses. Munich et al. revealed that dexosomes can stimulate caspase activity and result in tumor cell apoptosis via expressing the ligands of TNF superfamily such as TNF, FasL and TRAIL on their external surface [129]. Consistent with these findings, a study demonstrated that hyperthermic CO_2_-treated dexosomes were able to suppress proliferation and trigger apoptosis to a certain extent in gastric cancer cells [130]. Moreover, dexosomes were shown to induce propagation of splenic cells and promote cytotoxic ability against L1210 tumor cells [131].

### Dexosomes as potential cell-free tools for cancer immunotherapy

Dexosomes represent promising antitumor entities because of their potent immunostimulatory effects, their insensitivity to the immunosuppressive tumor microenvironment, and their potency to reduce tumor burden in laboratory models. In the following section, we will focus on the ability of dexosomes to initiate effective innate and adaptive immune responses in preclinical models.

#### Dexosome-mediated innate immune responses

Accumulating evidence have demonstrated that dexosomes mediate interactions that result in stimulation of cells of the innate immune system. Of note, in addition to MHCs, dexosomes carry proteins that are able to trigger or inhibit immune recalls in an antigen-independent manner. Dexosomes can stimulate NKs by providing ligands that bind to NK-activating receptors. This process is either mediated by dexosomal HLA-B-associated transcript 3 (BAT3; BAG6 (BCL2-associated athanogene 6); a ligand for natural cytotoxicity triggering receptor 3 (NCR3)) [132] or by UL16-binding molecules, i.e. MHC I polypeptide-related sequence A and B (MICA and MICB; ligands of NKG2D (natural killer group 2 member D)), on dexosomes [133]. In a murine model of advanced melanoma, dexosomes were shown to induce IL15Rα and NKG2D-dependent proliferation of NKs and promote IFNγ release which result in antimetastatic effects of NKs within the local tumor environment [134]. In human melanoma, dexosomes expressing NKG2D ligands on their surface could directly interact with NKG2D and NKs, supporting the hypothesis that dexosomes are capable of stimulating antimetastatic immune responses through a non-MHC-dependent manner. Similar to DCs, dexosomes can affect NKs to produce IFNγ by the interaction of dexosomal TNF with TNF receptor on NKs [129]. Dexosomes also express TLR4 (Toll-like receptor 4) and TLR1/2 ligands on their surface which cause enhanced expression of TNF and subsequent activation of NKs [135]. These findings describe novel characteristics of dexosomes in regulation and elicitation of NK-related immune responses, and propose new approaches for evaluating dexosome-mediated antitumor efficacy.

#### Dexosome-mediated adaptive immune responses

1. CD8^+^ T cells.

Several studies revealed that dexosomes have the potential of activating CD8^+^ T cell clones in vitro either alone [136] or when incubated with DCs that produce allogeneic MHC I proteins [137]. These findings indicate functionality of dexosomal antigen-MHC I assemblies. The first evidence supporting dexosome-mediated triggering of CD8^+^ T cells was reported in 1998 by Zitvogel and colleagues [107]. They showed that dexosomes isolated from BMDCs containing TAA-MHC I complexes acted efficiently in both suppression and eradication of an established malignancy in immune-competent but not immune-deficient mice [107]. The efficiency of this process was improved when dexosomes were administered concomitantly with mature DCs or chemical adjuvants that encouraged DC maturation [118]. Zitvogel et al. also demonstrated that dexosomes were more effective than their parent DCs in terms of tumor suppression, and that autologous (not allogeneic) dexosomes could induce TAA-targeted CTL response ex vivo, emphasizing the role of dexosomal MHC I throughout this process [107]. Dexosomes of mature DCs (in comparison with the dexosomes of immature DCs) were more effective in triggering CD8^+^ T cell immunity, indicating the importance of costimulatory factors present on dexosomes of mature DC origins [138]. Another study demonstrated that human dexosomes loaded directly with MART1 peptide (melan-A antigen; a TAA) harbored intact functional peptide-MHC I assemblies to target DCs ex vivo [118]. Here, it was further shown that dexosome-pulsed DCs were more effective in activation of CD8^+^ T cells than peptide-pulsed DCs. Induction of CD8^+^ T cell recalls was also confirmed when DCs lacking the TAP molecules (transporter associated with antigen processing) were used as the recipient [139]. In endocytic pathway, internalized antigens are carried from endosomes into the cytosol for proteasomal degradation [140]. Antigen-derived peptides are then carried by the TAP molecules into the endoplasmic reticulum or back into the antigen-containing endosomes, where they can be incorporated onto MHC I molecules [141]. However, Lawand et al. recently reported that some antigens may enter the endocytic pathway in a TAP-independent manner, indicating the possibility of other transporters dedicated to antigen translocation into endosomes for cross-presentation to CTLs [139].

Dexosome-mediated transference of peptide-MHC I assemblies to DCs was also observed in vivo. Autologous dexosomes loaded with antigenic peptides were able to transfer them to allogeneic DCs and initiate peptide-specific stimulation of CD8^+^ T cells in mouse. Intriguingly, intravenous administration of autologous dexosomes alone did not trigger any CD8^+^ T cell response [118]. Likewise, no noticeable level of antigen-specific CD8^+^ T cells was observed when MHC I-restricted peptide of ovalbumin (OVA, SIIN-FEKL) was loaded onto dexosomes [142]. Conversely, whole OVA protein-loaded dexosomes (with indirect method) were capable of initiating protein-targeted CD8^+^ T cell response. This effect depended mostly on CD4^+^ T cells and partly on B cells, particularly marginal zone B cells [142]. Further investigation by Hao et al. revealed that CD8^+^ T cell propagation induced by dexosomes loaded with protein relied on CD4^+^ T cells [143] and CD11c^+^ DCs [144].

A number of strategies was adopted in order to promote dexosomal-mediated antigen-specific CD8^+^ T cell responses. Viaud et al. introduced IFNγ as a key cytokine that stimulates dexosomal expression of CD40, CD80, CD86, and CD54 molecules which result in induction of direct and powerful antigen-dependent CD8^+^ T cell responses by dexosomes derived from IFNγ-matured MCDCs [111]. Another strategy is to inject dexosomes comprising a danger signal like a TLR ligand, such as polyinosinic:polycytidylic acid (poly (I:C)) or CpG-ODN, that boost DC maturation [118]. Here, a humanized MHC I-deficient murine model was used for administration of two therapeutic dosages of dexosomes pulsed with human peptides and coinjected with CpG-ODN, and it was found that tumor development was significantly decreased compared to mice that received tumor peptide-CpG-ODN [118]. αGC-loaded dexosomes were also reported to promote CD8^+^ T cell responses against a concurrently loaded antigen [145]. When DCs were exposed to the lysates of B16F10 melanoma cells with the aim of loading TAAs onto dexosomes, the prepared vaccine led to stimulation of melanoma-specific CD8^+^ T cells and recruitment of CTLs, NKs, and NKTs in the subcutaneously grafted melanoma tumors in mice. Consequently, tumor development was remarkably decreased and survival prolonged. DCs loaded with gastric TAAs demonstrated the ability to trigger the proliferation of CTLs. Additionally, by binding to TLR ligands, dexosomes can activate adjacent DCs to express transmembrane TNF and produce pro-inflammatory cytokines [146].

Several investigations attempted to change the molecular make-up of dexosomes to generate tolerogeneic vesicles with immunosuppressive features. Genetically-modified BMDCs produce IL4, IL10 or FasL molecules that repress the inflammation caused by DTH in a murine model of collagen-induced arthritis [147, 148]. Similarly, when donor dexosomes were injected to a rat model of cardiac transplantation, chronic allograft rejection response was remarkably delayed [149]. Dexosomes produced from TGFβ1- and IL10-matured DCs could also induce immune tolerance in a skin allograft murine model [150]. Moreover, DCs overexpressing indoleamine 2,3-dioxygenase (IDO) molecules produced dexosomes that reduced inflammation in a rheumatoid arthritis model [151]. Lu et al. recently demonstrated that in a murine model of autochthonous hepatocellular carcinoma, mice treated with dexosomes isolated from DCs that expressed α-fetoprotein (AFP) had remarkably more IFNγ-producing CD8^+^ T cells, enhanced levels of IL2 and IFNγ, fewer Tregs and reduced levels of IL10 and TGFβ [152]. Therefore, dexosomes are capable of promoting T cell stimulation along with downregulating immunosuppressive responses, which serve as a promising tool to create efficient antitumor vaccines.

2. CD4^+^ T cells.

While dexosomes can present antigens and directly trigger cognate CD8^+^ T cell clones, lines [138], or primed CD4^+^ T cells [153], they need to be captured by bystander DCs to activate naïve CD4^+^ T cells [91, 125, 153]. Dexosomes were reported to transfer peptide-MHC II complexes to MHC II-deficient DCs, and allowed them to trigger antigen-specific CD4^+^ T cells [91, 125]. As reported for CD8^+^ T cells, dexosomes of mature DCs were also more effective in CD4^+^ T cell activation in vitro [91]. Peptide- or protein-loaded dexosomes could hinder tumor growth by recruiting CD4^+^ and CD8^+^ T and B cells in vivo [142]. Further in vivo studies demonstrated that dexosomes loaded with antigens and generated using TLR3 agonist, poly (I:C), and OVA initiated the propagation of OVA-specific CD4^+^ and CD8^+^ T cells [154]. These results were remarkably superior compared to using CpG-B and LPS for TLR9 and TLR4, respectively. When antigen-pulsed dexosomes from mature, but not immature, DCs were injected from male into female mouse models, the male skin grafts were rejected because activated CD4^+^ T cells were differentiated into effector CD4^+^ T cells in vivo [91].

As previously mentioned for CD8^+^ T cells, dexosomes are able to initiate propagation of antigen-specific CD4^+^ T cells once they are loaded with whole protein antigens. It was assumed that this effect depended upon a functional compartment belonging to B cells, since the proliferation of CD4^+^ T cells was not detected in B cell receptor signaling deficient btk^−/−^ mice [155]. These findings imply that dexosome-borne antigens are ingested, processed, and presented by DCs to T and B cells, a hypothesis further approved in an investigation where allogeneic I-Ad^+^ dexosomes could stimulate allo-specific CD4^+^ T cell proliferation in I-Ab^+^ mice [121]. Dexosomes loaded directly or indirectly with peptide-MHC II assemblies could trigger specific CD4^+^ T cell responses in vivo [121, 125]. In vitro, dexosomes could not trigger antigen-specific T cell activation unless mature CD8α^−^ DCs were also present in the culture environment. These mature DCs could be MHC II-deficient, but had to express CD80 and CD86 costimulatory factors. Moreover, in comparison with CD8α^+^ DCs, CD8α^−^ DCs were more powerful in induction of CD4^+^ T cell immunity [125]. In another study, it was reported that CD8α^+^, but not CD8α^−^, DCs purified from the lymph nodes of dexosome-treated mice were capable of stimulating antigen-targeted CD4^+^ T cell proliferation ex vivo [121, 156]. Qazi et al. compared the efficiency of dexosomes directly loaded with OVA peptide with dexosomes derived from OVA-pulsed DCs in initiating specific CD4^+^ T cell responses in vitro and in vivo, and showed that both dexosomes could elicit T cell proliferation in vitro, with peptide-loaded dexosomes being more effective. Conversely, in vivo, only dexosomes produced from OVA-pulsed DCs could induce CD4^+^ T cell proliferation, emphasizing the significance of indirect antigen-loading approaches in clinical applications. Moreover, these dexosomes were able to induce the polarization of T cells to the Th1 type in a B cell-dependent fashion, which highlights the importance of B cells in producing T cell responses through a dexosome-dependent pathway [155].

3. B cells

Exosomes originated from various APC origins contribute to the elicitation of B cell immunity both ex vivo and in vivo. Segura et al. demonstrated that dexosomes can harbor both antigen-MHC assemblies and ICAM1 molecules to less-efficient APCs, such as B cells, leading to T cell stimulation in an indirect way [91]. Naslund et al. also demonstrated B cells were essential for optimal triggering of CD8^+^ T cells via dexosomes [157]. Mycoplasma-infected BMDCs release dexosomes that are able to initiate polyclonal propagation of primary B cells independent of CD40, LPS, or CpG signaling pathways in vitro [158]. In another study, allogeneic BMDC dexosomes were administered systematically (intravenous/intraperitoneal injection) to rats before transplantation with the aim of exploring anti-allograft immune responses. Here, dexosomes could initiate the in vivo production of IgG2a and b antibodies (type I antibodies) specific to dexosomal antigens, and resulted in extended survival of the allograft [159]. Likewise, BMDCs pulsed with diphtheria toxoid or OVA antigen resulted in the production of antigen-specific type I antibodies [155, 160]. Titers of specific antibodies can be amplified by simultaneous pulsing of BMDCs with αGC and an antigenic protein [145].

Taken together, all the above-mentioned reports confirm that dexosomes are perfectly capable of exerting potent immune responses and hence possess great therapeutic values against a variety of immune-related diseases, including malignancies.

### Dexosome-based cancer immunotherapy in clinical trials

**Fig. 3:**
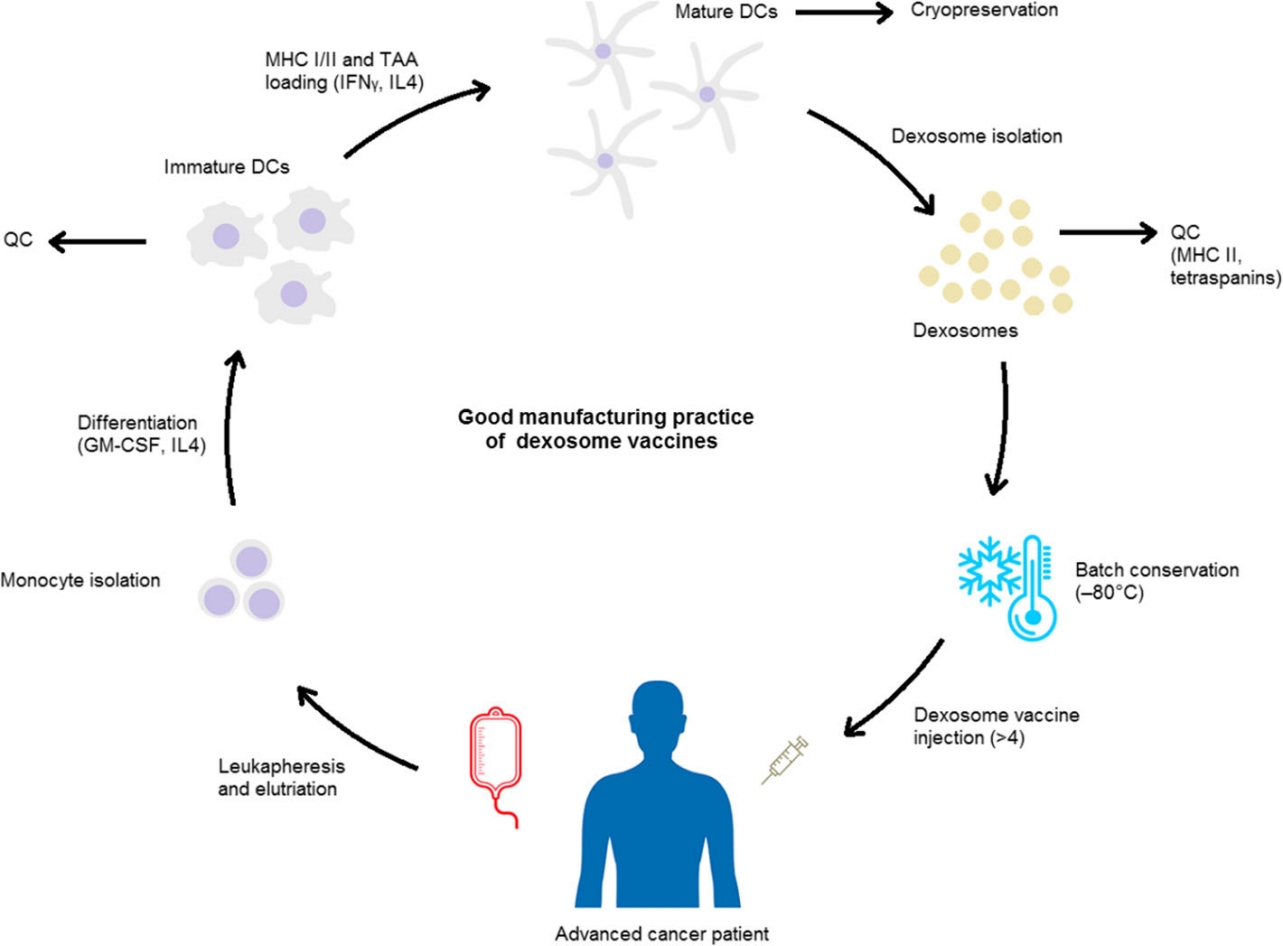
The good manufacturing practice of dexosome cancer immunotherapy. Advanced cancer patient is first subjected to leukapheresis. Following elutriation, monocytes are isolated in a cell therapy unit good manufacturing practice laboratory, and are then differentiated into immature dendritic cells (DCs) using GM-CSF and IL4, and may go through a quality control (QC) check as well. Afterwards, MHC I and II molecules incorporated with tumor-associated antigens (TAAs) are loaded onto DCs in the presence of IFNγ. TAA-loaded dexosomes are then produced and can be collected from culture supernatants. Each dexosome preparation is checked for immunological features (e.g., the content of tetraspanins, MHC II and costimulatory molecules) and immunostimulatory potential (e.g., the capacity to trigger a cognate T cell clone) before releasing of a certain batch. Released batches may subsequently be cryopreserved (at − 80 °C) for future administrations. Manufacturing of dexosome vaccines for cancer immunotherapy requires roughly three weeks following leukapheresis (this involves the time for in vitro cell culture steps, production and isolation of dexosomes, QC checks, and first-line therapy of the patient with metronomic cyclophosphamide)

In light of their great capacity as immunotherapeutic agents, dexosomes have been employed as cell-free anticancer vaccines in several clinical trials, including two phase I [161, 162] and one phase II [163] clinical trials in end-stage cancer patients. Since preparation of dexosome vaccines is possible using cells isolated from a single step of leukapheresis, hundreds of individual dexosome vaccine preparations have been created thus far. The process of good manufacturing practice of dexosome cancer vaccines is represented in Fig. 3. Quality control criteria for the produced vaccines include designation of tetraspanin proteins (CD63, CD81, and CD82) as well as the overexpression of HLA-DR and other dexosomal hallmarks including Tsg101 and HSP70 [111]. Loading of TAAs on dexosomes was verified by pulsing dexosomes with or without HLA-A2^−^ DCs and an antigen-specific T cell clone. In the phase II trial, manufacturing of dexosome batch relied on the increased proportion of tetraspanin and HLA-DR (MHC II) proteins in comparison with the control [111, 163]. While natural epitopes of dexosomal MHC I proteins were eluted using acid in the phase I trials, no precedent elution was conducted in the phase II trial. This alteration in elution strategy was due to the ability of peptides with higher affinity to coincide with or compete against the natural epitopes present on dexosomal membrane, as was demonstrated by in vitro functional tests utilizing MART1-specific CTL clones [111, 163].

#### Phase I clinical trials

Autologous MCDC cultures loaded with HLA-elicited MAGE-A3, -A4, -A10, and MAGE-3DPO4 peptides (melanoma-associated antigens) were employed in the primary phase I studies. In both trials, four doses of dexosome vaccinations were administered at weekly intervals. Thirteen HLA-A2^+^ participants with pretreated final stage (IIIb and IV) non-small cell lung carcinoma (NSCLC) overexpressing MAGE-A3 or -A4 antigens were qualified to receive dexosome immunotherapy in the first line of phase I trials [161], and nine participants completed the therapy. One week following the last vaccination, three of the tested patients, who had not shown MAGE-sensitive immune responses before immunization, exhibited systemic MAGE-specific immune reactivity as confirmed by DTH response. Increased MAGE-specific T cell function was only observed in one of five patients, as determined by enzyme-linked immunospot (ELISPOT) assay. This low-level T cell reactivity was attributed to the possible suppression of Tregs (CD4^+^ CD25^+^ T cells). In two of three participants, Tregs were amplified (compared to the baseline levels) as a percentage of total CD4^+^ T cells following dexosome therapy. An improvement of the NK lytic activity was detected in two of four tested samples. Taken as a whole, the NSCLC phase I trial was well tolerated and showed an acceptable safety profile, with disease stability shown in two participants who had progressive tumors at diagnosis. Additionally, disease stability continued for over twelve months in two of four participants who had initially stable disease [161].

In the second study of phase I trials, fifteen participants with the following criteria were enrolled: stage IIIb/IV, HLA-A I^+^ or HLA-B35^+^ and HLA-DPO4^+^ leukocyte phenotype, MAGE-3-overexpressed malignant melanoma (MM) [162]. The MCDC dexosome administration format included four vaccine doses at weekly intervals. Two dosages of either MHC II proteins (0.13 and 0.40 × 10^14^ molecules) or peptides (10 and 100 µg/mL) were administered. Evaluation of the therapy efficiency was conducted two weeks postimmunization. One of the patients showed a partial reactivity to dexosome therapy. In this patient, a depigmentation halo surrounding naevi was observed, and the arterial neovasculature was disappeared and tumor retracted. This participant received four months of continuation therapy with dexosomes which resulted in the disease stabilization and toxicity reduction. Stabilization of the disease for up to twenty four months was too observed in another participant who was given continued dexosome therapy. Taken together, this study resulted in two stabilized diseases along with one minor, one partial, and one mixed reactivity at lymph nodes or skin. A number of these results were achieved in participants with invasive tumors who had formerly gone through other therapies or received alternate anticancer vaccines. As observed in the first phase I trial, neither MAGE-specific CD4^+^ and CD8^+^ T cell activation nor DTH response were identified in the peripheral blood of patients [162]. However, it was demonstrated that immature MCDC dexosomes express NKG2D ligands on their membrane which bind to NKG2D on NKs, resulting in pro-NK effects [134]. After four weeks of vaccine administration, the number of circulatory NKs were remarkably enhanced. After dexosome immunotherapy, the expression of NKG2D and NK cytotoxicity were maintained in 50% of patients who had NK function deficit at the beginning of the study [134]. Moreover, it was found that dexosome therapy could trigger NK proliferation in vivo in an IL15Rα-dependent fashion. These findings were in consistence with the improved control of tumor metastasis in B16F10 melanoma cell-inoculated mice by NK1.1^+^ cells [134]. Human dexosomes derived from immature DCs were reported to present BAG6 molecules on their surface [164], which is a ligand for NKp30 receptors expressed on NKs [165]. Cytokine release from NKs was reported to be directly correlated with the expression levels of BAG6 on dexosomes [164]. Furthermore, dexosomal surface expression of TNF were able to trigger the production of IFNγ by NKs [129].

#### Phase II clinical trials

Defective antitumor effects of first-generation dexosomes (IFNγ-free dexosomes) in the induction of NKs and T cells in the phase I trials encouraged researchers to design and develop innovative strategies in order to promote dexosome-dependent antitumor host immune responses. As mentioned before, one strategy is to utilize dexosomes originated from LPS- or IFNγ-matured DCs, which exhibit greater T cell immunity [111]. A clinical-grade process for production of dexosome vaccines was developed when these results were applied to human DC cultures [111]. Here, IFNγ was employed for stimulating human DCs in culture, and subsequently, dexosomal costimulatory factors and ICAMs were upregulated, resulting in second-generation dexosomes (IFNγ dexosome) with increased immunostimulatory capacity [111, 166]. The phase II clinical trial aimed to investigate whether maintenance immunotherapy of advanced NSCLC patients using IFNγ dexosomes could result in progression-free survival (PFS) at four months postchemotherapy [163]. Twenty two HLA-A2^+^ patients who had inoperable (stage IIIb or IV) NSCLC with neutrophils ≥ 1.5 × 10^9^/L and showed immune responses or disease stabilization following four rounds of a first-line platinum-based chemotherapy were qualified for receiving IFNγ dexosome [163]. The utilized TAAs included MAGE-A1, -A3, NY-ESO, MART1 (all MHC I-restricted peptides) and EBV (MHC II-restricted peptides). Patients first received three weeks of metronomic oral low-dose cyclophosphamide (CTX). This regimen was proven to decrease Treg activity and induce IFNγ- and IL17-generating T cell clones according to several preclinical [167–169] and clinical investigations [168, 170]. Therefore, dexosome-mediated priming of T cells is facilitated, and NK and T cell functions are preserved. Of these participants, seven patients (32%) showed disease stability following nine times of dexosome vaccination, and proceeded to receive the therapy at three-week intervals. Unfortunately, the main clinical outcome of the phase II trial, a PFS of 50%, could not be achieved and no remarkable immune reactivity was reported in the study. Despite loading of multiple epitopes and CTX adjuvant therapy, the use of IFNγ dexosomes was insufficient to manifest TAA-specific T cell reactivity [163]. However, one participant elicited a long-term disease stabilization which allowed for surgical removal of the tumor and the eligibility for local adjuvant radiotherapy. Moreover, second-generation dexosome therapy was reported to promote NKp30-mediated NK function. Although downregulated in stage IV NSCLC, NKp30 activation improved the release of TNFα and IFNγ by blood NKs, which was detected after four cycles of dexosome vaccination. More significantly, this augmented NKp30-mediated NK function was associated with longer PFS [163]. Moreover, the aforementioned membrane-associated NKp30 ligand, BAG6, was detected on the membrane of dexosome vaccine preparations and was reported to be responsible in NKp30-dependent pro-NK activity. To further support this theory, it was shown that the concentration of MHC II molecules in dexosome inocula and NKp30-dependent NK activity correlated with the levels of dexosomal BAG6. This is different from the results obtained in MM phase I trial where NKG2D (and possibly IL15 or IL15Rα) mediated dexosome-related NK responses [134, 162]. Since dexosome preparations employed in the phase I MM study were not obtained from IFNγ-matured DCs (where IFNγ results in BAG6 overexpression [171]), the NKG2D ligand-mediated NK activation was probably manifested more dominantly in the absence of BAG6-NKp30 pathway.

### Application of distinct subsets of DCs to ameliorate cancer immunotherapy

The unique characteristics of diverse DC subsets can be employed to generate more potent and efficient antitumor immune responses. Since DCs are hardly present in the peripheral blood (approximately 1% of total leukocytes), most of DC-based vaccine preparations are derived from moDCs differentiated in vitro from CD34^+^ progenitors or CD14^+^ monocytes by the addition of growth factors (such as IL4 and GM-CSF) and maturation factors (e.g. CD40L or TLR agonists) and subsequently loaded with antigenic material [172–175]. However, ex vivo differentiated moDCs differ from the primary circulatory DCs both in phenotypic and transcriptional features [176] and are less efficient in migratory capacity and T cell activation [177, 178]. Recent investigations in cancer immunotherapy recommend using autologous DCs and focus on DC subset specificity [179]. Different subsets of DCs are probably correlated with improved survival rate in various tumor types [29, 36, 40]. Given the assumption that all subsets of DCs are capable of triggering antitumor immunity, a number of clinical trials were performed [180–182] utilizing autologous primary pDCs and cDC2s [183]. Although cDC1s remain the most effective candidate against cancer, according to their potent CD8^+^ T cell activation potential, their separation from the peripheral blood is still a major challenge for their clinical use [183]. Flt3L-dependent mobilization can be used for stimulation of all DC subsets, including cDC1s, in vivo [180, 182]. However, ongoing attempts are investigating new approaches for optimization of cDC1-based vaccines for application in clinical setting. Recently, the role of the Notch signaling route was proposed in promoting in vitro differentiation of cDC1s [184, 185]. An alternative and promising strategy is to directly reprogram dermal fibroblasts into cDC1s [186]. The effectiveness of cDC1-based cancer vaccines was tested in several experimental models and the results will pave the way for their application in clinical trials in the near future [187].

Regardless of the recent breakthroughs in immunotherapy of cancer, manipulation of DCs or their EVs for development of more effective vaccine tools has not yet attained its maturity, and the optimization of mechanisms at subcellular and molecular level is still in demand for promoting their antitumor effects. It was shown that cellular maturity in cDC1-based vaccines can be reached by stimulating TLR3 (poly I:C) and TLR8 (R848) [188, 189]. The method of antigen loading is a major factor governing the priming of T cells. For instance, in a personalized clinical trial of ovarian cancer [190], moDCs incubated with lysates of autologous oxidized whole cancer cells extended survival. Another strategy is to use engineered DCs with a chimeric receptor that can exclusively uptake TAA-bearing EVs and lead to efficient antitumor immune responses [191]. Processing and cross-presentation of antigens can be promoted by regulating the proteolysis of internalized antigens through the abrogation of molecules regulating vesicular trafficking including YTHDF1 and SEC22B [27, 192]. Alternatively, to generate potent T cell responses, the cDC1-intrinsic immunosuppressive signals can be silenced using siRNA molecules that delete PDL1 and 2 [193]. The synergic application of immune checkpoint blockade and DC-based vaccines promises one of the most efficient approaches for cancer immunotherapy. Another promising strategy is to promote the cross-presentation potential of cDC1s by directly targeting CLEC9A, which contributes to internalization of dead cells and presentation of antigenic material to CD8^+^ T cells [194, 195]. Anti-CLEC9A molecules can deliver maturation signals to cDC1s as recently demonstrated by infusion of a chimeric recombinant protein inside the tumor mass [196]. Chemokines that regulate the employment of cDC1s in tumors can also be targeted to promote the clinical efficacy of DC-based vaccines as was demonstrated using intratumoral injection of CCL21-expressing DCs to trigger antitumor immune responses in a phase I lung cancer clinical trial [197]. Targeting XCR1 was also demonstrated to be involved in TAA delivery to cDC1s and subsequent activation of CD8^+^ T cells [198, 199]. Next-generation DC-based vaccines, including dexosome vaccines, will most probably recruit the above-mentioned strategies to promote the differentiation of cDC1s ex vivo and utilize their dexosomes for generating efficient therapeutic responses.

## Conclusions

The idea of employing dexosomes as anticancer vaccine vehicle is utilizing the nature’s antigen delivery system for vaccination. However, the low clinical efficacy of dexosome vaccines in induction of adaptive immune responses can be explained by the advanced stage of the disease, the limited number of patients, who had received antitumor therapies prior to enrollment, and the lack of appropriate preselection criteria [200]. According to the phase II trial data [163], dexosome immunotherapy was probably most effective in patients with measurable levels of serum BAG6, which is possibly related to NKp30 functional defects. In other words, patients who showed downregulation or defective functions of NK receptors (particularly NKG2D or NKp30) were most likely to benefit from dexosome therapy [163]. The presence of local or systemic immunoregulatory mechanisms such as the expression of PDL1 on NSCLC cells in association with PD1 overexpression in myeloid-derived suppressor cells, Tregs and tumor-infiltrating lymphocytes is another possibility for the observed poor immune reactivity. Another conceivable explanation is that the utilized dexosome MHC I/II-restricted TAAs were not sufficient to stimulate tumor-targeted T lymphocyte reactivity. Moreover, the pharmacokinetic of the injected dexosomes is not yet fully determined. Although it is expected that the injected dexosomes reach T cell zones of secondary lymphoid organs in acceptable quantities, they may have travelled to the macrophages of subcapsular sinus or DCs of lymphatic sinus where they interact with innate lymphocytes [201].

Dexosome vaccines function more successfully when combined with other therapy regimes. For instance, in CTX-exosome combination therapy, CD8^+^ T cell priming against the preestablished tumor was synergistically increased in mice [167]. However, in humans, CTX-dexosome combination chemotherapy appeared to be efficient only if genuine adjuvants were present. The therapeutic outcome observed here is probably dependent on the repression of tumor-elicited tolerance along with promotion of tumor-mediated immunogenicity, since CTX is capable of alleviating Treg function [167, 170]. This CTX-mediated strategy was implemented in the phase II trial [163, 166], although it is presumed that the relevant clinical outcome would have been more successful in earlier stages of disease. Another proposed combination therapy is to join PD1/PDL1 blocking (or coblocking with anti-CTLA4 therapy) with dexosome vaccine, which results in the suppression of tumor-infiltrating lymphocytes and T cell activation. However, one major obstacle is that the presently established regimen for chemotherapy of NSCLC is not able to trigger immune-related cellular death [202]. Therefore, combination therapy utilizing immunogenic cytotoxic drugs poses an appropriate option in treatment of malignancies. A last conceivable option is to combine dexosome vaccine with NK-based therapies, such as anti-KIR Ab (anti-killer cell immunoglobulin-like receptor antibody) [203], in order to generate synergistic immunogenic results against NK-dependent cancers. Furthermore, establishing an immortalized DC line library, tailored for expression of a single MHC I and/or II allele or no MHC proteins, will make continuous production of dexosomes possible, and decrease therapy expenses and the delay caused by extended culture times. Despite the obstacles hurtled thus far, the vision of dexosome cancer immunotherapy still stands highly promising, offering multiple benefits over living cell transplantation. Thus, one can envision that dexosomes might replace cell-based therapeutic strategies in the long run.

## Data Availability

Not applicable.

## Abbreviations

AFP: α-fetoprotein
Anti-KIR Ab: Anti-killer cell immunoglobulin-like receptor antibody
APC: Antigen-presenting cell
BAG6: BCL2-associated athanogene 6
BAT3: HLA-B-associated transcript 3
BDCA2: Blood dendritic cell antigen 2
BMDC: Bone marrow-derived dendritic cell
CAM: Cell adhesion molecule
CCL22: C-C motif chemokine 22.
CCR7: C-C chemokine receptor type 7
cDC: Conventional DC
CLEC9A/ DNGR1: C-type lectin domain family 9 member A
CTL: Cytotoxic T lymphocyte
CTX: Cyclophosphamide
CpG-ODN: CpG oligodeoxynucleotide
CXCL9: C-X-C motif chemokine 9
CXCR3: C-X-C chemokine receptor type 3
DAMP: Damage-associated molecular pattern
DC: Dendritic cell
DTH: delayed-type hypersensitivity
DUB: deubiquitinating enzyme
ELISPOT: enzyme-linked immunospot assay
ESCRT: endosomal sorting complexes required for transport
EV: extracellular vesicle
FasL: Fas ligand
Flt3L: FMS-like tyrosine kinase 3 ligand
GM-CSF: Granulocyte-macrophage colony-stimulating factor
HLA: Human leukocyte antigen
HSP: Heat shock protein
ICAM1: Intercellular adhesion molecule 1
ID2: DNA-binding protein inhibitor ID-2
IDO: Indoleamine 2,3-dioxygenase
IFN: Interferon
IL: Interleukin
IL15Rα: Interleukin 15 receptorα
ILV: Intraluminal vesicle
iNKT: Invariant natural killer T cell
IRF8: Interferon regulatory factor 8
KLF4: Krueppel-like factor 4
LITAF: Lipopolysaccharide induced TNF factor
LFA1: Lymphocyte function-associated antigen 1
LPS: Lipopolysaccharide
MCDC: Monocyte-derived dendritic cell
MART1: Melan-A antigen
mCRPC: Metastatic castration-resistant prostate cancer
MFGE8: Milk fat globule-EGF factor 8
MHC: The major histocompatibility complex protein
MICA: MHC I polypeptide-related sequence A
MICB: MHC I polypeptide-related sequence B
MM: Malignant melanoma
moDC: Monocyte-derived DC
MVB: Multivesicular body
NCR3: Natural cytotoxicity triggering receptor 3
NK: Natural killer cell
NKG2D: Killer cell lectin-like receptor subfamily K, member 1 ligands
NSCLC: Non-small cell lung carcinoma
OVA: Ovalbumin
OX40L: OX40 ligand
PAMP: Pathogen-associated molecular pattern
PAP: Prostatic acid phosphatase
pDC: Plasmacytoid DC
PD1: Programmed cell death protein 1
PDL1: Programmed cell death protein ligand 1
PFS: Progression-free survival
poly (I: C): Polyinosinic:polycytidylic acid
SIMPLE: Small integral membrane protein of the lysosome/late endosome
siRNA: Small interfering RNA
SNARE: Soluble N-ethyl maleimide (NEM)-sensitive factor attachment protein receptor
STAM: Signal transducing adapter molecule
TAA: Tumor-associated antigen
TAP: Transporter associated with antigen processing molecule
TCR: T cell receptor
TGFβ: Transforming growth factorβ
Th: Helper T cell
TLR: Toll-like receptor
TNF: tumor necrosis factor
TRAIL: TNF-related apoptosis-inducing ligand
Treg: T regulatory cell
TSAP6: Transmembrane protein tumor suppressor-activated pathway 6
ULBP1: UL16-binding protein 1
Vps32: Vacuolar protein sorting-associated protein 32
XCL1: XC chemokine ligand 1
XCR1: Chemokine XC receptor 1
ZEB: Zinc finger E-box-binding homeobox
